# Pharmacologic Resistance in Soft Tissue Sarcomas: Mechanisms, Biomarkers, and Translational Therapeutic Strategies

**DOI:** 10.3390/cancers18142364

**Published:** 2026-07-22

**Authors:** Dorian Yarih García-Ortega, Gabriela Alamilla-García, Kevin Fernando Reyna-Pérez, Jessica Baldriche-Acosta, Luis Alonso Herrera-Montalvo, Carlo César Cortés-González

**Affiliations:** 1 Departamento de Cirugía Oncológica, Instituto Nacional de Cancerología, Ciudad de Mexico 14080, Mexico; dr_doriangarcia@me.com; 2 Departamento de Oncología Médica, Instituto Nacional de Cancerología, Ciudad de Mexico 14080, Mexico; gabync28@hotmail.com (G.A.-G.); freynap@gmail.com (K.F.R.-P.); 3 Secretaría del Medio Ambiente de la Ciudad de México (SEDEMA), Ciudad de Mexico 06060, Mexico; jessicabaldriche@gmail.com; 4 Instituto Nacional de Ciencias Médicas y Nutrición Salvador Zubirán, Ciudad de Mexico 14250, Mexico; 5 Escuela de Medicina y Ciencias de la Salud, Tecnologico de Monterrey, Ciudad de Mexico 14380, Mexico; 6 Unidad de Investigación Biomédica en Cáncer, Instituto Nacional de Cancerología, Ciudad de Mexico 14080, Mexico

**Keywords:** soft tissue sarcoma, drug resistance, tumor microenvironment, biomarkers, immunotherapy, targeted therapy, liquid biopsy, precision oncology

## Abstract

Soft tissue sarcomas are rare, biologically diverse mesenchymal malignancies in which pharmacologic resistance cannot be explained by a single unifying mechanism. In this narrative review, resistance is conceptualized as a dynamic, multilayered process shaped by histologic subtype, genomic architecture, transcriptional plasticity, the tumor microenvironment, and treatment-driven selective pressure. Resistance to conventional chemotherapy arises through both intrinsic and acquired mechanisms, including altered drug transport and metabolism, enhanced DNA damage responses, impaired apoptotic signaling, clonal selection, and the emergence of therapy-persistent cellular states. By contrast, resistance to targeted and epigenetic therapies more often reflects adaptive bypass signaling, lineage reprogramming, and incomplete identification of subtype-specific dependencies than secondary on-target alterations alone. The tumor microenvironment further contributes to therapeutic failure through hypoxia, extracellular matrix-mediated barriers, abnormal vascularization, myeloid-dominant immunosuppression, and immune exclusion, thereby helping explain the modest and histology-dependent activity of immune checkpoint inhibitors in soft tissue sarcoma. This review also differentiates baseline predictive biomarkers from dynamic resistance-monitoring tools, underscoring the potential—despite still limited clinical maturity—of pharmacogenomic markers, immune signatures, tertiary lymphoid structures, circulating tumor DNA, and circulating methylation-based approaches. Finally, emerging strategies to overcome resistance are examined, including mechanism-based combinations, biomarker-guided treatment selection, synthetic lethality, functional precision platforms, and adaptive histology-specific trial designs. Collectively, these observations support a view of resistance in soft tissue sarcoma as a context-dependent biological process that demands integrated, subtype-aware, and translationally grounded therapeutic strategies.

## Simple Summary

Soft tissue sarcomas are a rare and highly diverse group of cancers that often become resistant to treatment, making them difficult to control. Because these tumors differ greatly in their biological characteristics, resistance cannot be explained by a single cause. This review aims to summarize the many factors that contribute to treatment failure, including genetic alterations, changes in tumor cell behavior, interactions with the surrounding tumor environment, and the effects of previous therapies. It also discusses current and emerging biomarkers that may help predict treatment response and monitor resistance over time. In addition, the review highlights new strategies designed to overcome resistance, such as biomarker-guided therapies, combination treatments, and precision medicine approaches tailored to specific sarcoma subtypes. By providing an integrated overview of these mechanisms, this work may support the development of more effective and personalized treatment strategies for patients with soft tissue sarcoma.

## 1. Introduction: Why Resistance in STS Requires Its Own Framework

Soft tissue sarcomas (STS) constitute a rare and heterogeneous group of mesenchymal malignant neoplasms. Rather than a single disease, they encompass dozens of clinicopathologic entities that differ substantially in histogenesis, morphology, genomic complexity, biological behavior, and therapeutic sensitivity. This diversity explains why the broad label “STS” groups tumors with markedly different biological behaviors and why overall results from clinical trials do not always accurately reflect the sensitivity or refractoriness of specific histotypes [1,2].

In advanced disease, therapeutic progress has been real but limited. Doxorubicin remains the backbone of first-line systemic treatment for most adult patients with advanced STS, and intensification with ifosfamide may increase response rates and promote greater tumor shrinkage, although it has not consistently translated into an overall survival benefit and carries greater toxicity [2,3]. Consistent with this, the GeDDiS trial did not demonstrate superiority of gemcitabine/docetaxel over doxorubicin as an initial strategy for unresectable or metastatic disease [4]. In later lines, targeted agents such as pazopanib have shown activity in selected subgroups, particularly in previously treated non-adipocytic STS, but their clinical benefits remain modest and do not uniformly alter the natural history of the disease [5]. Even promising early signals may fail to hold in confirmatory validation, as with olaratumab in the ANNOUNCE trial, illustrating how difficult it is to translate preliminary results into reproducible benefit in such a biologically diverse population [6]. Although most clinical resistance in STS remains treated as a post-treatment phenomenon, contemporary translational models emphasize that resistance is frequently preconfigured by intrinsic tumor biology, microenvironmental architecture, and immune escape programs before therapy begins [7].

This context justifies the need for a distinct conceptual framework for pharmacologic resistance in STS. In these tumors, resistance should not be understood solely as a late loss of clinical efficacy but as an emergent phenomenon arising from interactions among tumor lineage, genomic architecture, transcriptional plasticity, the tumor microenvironment, and treatment-induced selective pressure. A narrative review focused on mechanisms is especially relevant because it integrates evidence dispersed across histotypes, drugs, and biological levels while avoiding the misleading impression that all STS share a uniform program of therapeutic sensitivity or escape [1,2,5,6]. The scope of this review is limited to adult STS and excludes GIST. Although gastrointestinal stromal tumors (GIST) share a mesenchymal origin, they follow a distinct biological and therapeutic paradigm, driven by oncogenic alterations in KIT and PDGFRA and by patterns of sensitivity and resistance to tyrosine kinase inhibitors developed within disease-specific frameworks [2,8].

This narrative review was informed by a structured search of PubMed/MEDLINE, Embase, Scopus, and Web of Science Core Collection for articles published from January 1990 to March 2026. Search terms included combinations of “soft tissue sarcoma,” “drug resistance,” “pharmacologic resistance,” “chemotherapy resistance,” “doxorubicin,” “ifosfamide,” “trabectedin,” “targeted therapy,” “tyrosine kinase inhibitors,” “epigenetic therapy,” “immunotherapy,” “tumor microenvironment,” “biomarkers,” “pharmacogenomics,” “ctDNA,” “liquid biopsy,” “synthetic lethality,” “organoids,” “functional precision medicine,” and “antibody-drug conjugates.” Eligible publications included clinical trials, prospective or retrospective clinical cohorts, translational studies using human tumor material, molecular pathology studies, practice guidelines, and high-quality reviews relevant to resistance or response in adult non-GIST STS. Studies were prioritized according to the hierarchy of evidence: prospective clinical and biomarker-validation studies first, followed by clinical–translational investigations, then preclinical models. Pediatric-only sarcoma studies, GIST-focused reports, bone sarcoma studies without a directly transferable mechanistic contribution, conference abstracts without sufficient methodological detail, and studies not addressing therapeutic response or resistance were excluded. Mechanisms supported primarily by experimental models or extrapolated from non-sarcoma settings are explicitly identified as hypothesis-generating rather than clinically validated.

## 2. Molecular Heterogeneity as a Substrate of Resistance

In adult STS, tumor biology can be usefully organized into two broad genomic frameworks: on the one hand, sarcomas driven by relatively well-defined alterations, particularly recurrent gene fusions, and on the other, sarcomas with complex karyotypes, characterized by chromosomal instability, extensive gains and losses of genetic material, and multiple structural alterations. The first group includes entities such as synovial sarcoma, dominated by SS18::SSX fusions, and myxoid liposarcoma, defined by FUS::DDIT3 or, less frequently, EWSR1::DDIT3. In contrast, subtypes such as undifferentiated pleomorphic sarcoma, leiomyosarcoma, and dedifferentiated liposarcoma typically display complex genomic landscapes, with alterations involving TP53, RB1, and ATRX, and, in dedifferentiated liposarcoma, amplification of MDM2 and CDK4. This distinction is not merely taxonomic; it shapes how resistance emerges. In tumors dominated by a major oncogenic event, refractoriness may depend more heavily on transcriptional reprogramming, epigenetic context, and lineage-specific dependencies. In sarcomas with complex genomes, by contrast, resistance often arises against a backdrop of greater biological redundancy and broader subclonal diversification [9,10,11].

### Fusion Oncoproteins as Lineage-Encoded Resistance Programs

Fusion oncogenes should be viewed not only as diagnostic hallmarks, but also as lineage-encoding regulatory programs that can establish the epigenetic, transcriptional, and microenvironmental states in which resistance emerges. In synovial sarcoma, SS18::SSX globally retargets BAF chromatin-remodeling complexes and activates a disease-specific transcriptional program, providing a biologically plausible route by which a defining fusion can shape cell identity and therapeutic dependency [12]. This does not establish a direct, clinically validated fusion-specific predictor of anti-PD-1 resistance; rather, it supports subtype-specific longitudinal study of fusion-driven lineage states, antigen presentation, and immune context.

In myxoid liposarcoma, FUS::DDIT3 and the tumor microenvironment jointly regulate cell–cell and cell–extracellular-matrix programs, chromatin remodeling, immune-response-associated pathways, and metabolism in preclinical models [13]. These data provide a second example of a fusion that may influence the local ecological niche, but the evidence remains experimental. Direct longitudinal evidence that changes in fusion transcript or protein quantity drive resistance to chemotherapy, targeted therapy, or PD-1 blockade in STS is currently insufficient; such measurements should therefore be regarded as investigational rather than as clinical resistance biomarkers [12,13].

Intratumoral heterogeneity adds a second layer of complexity. Even within the same lesion, different regions may show meaningful variation in expression profiles, cellular composition, and clonal hierarchies, undermining the assumption that a single biopsy can fully capture tumor biology. Gene expression studies in STS had already shown that intertumoral heterogeneity exceeds intratumoral heterogeneity, but they also demonstrated that the latter exists and may be biologically significant. More recently, longitudinal evolutionary analyses have shown that sarcomas do not follow a single trajectory of progression: some preserve a relatively stable driver axis for years, whereas others accumulate clonal branching and progressive genetic diversification. From a therapeutic perspective, this means that an initial response may reflect the sensitivity of the dominant clone, whereas relapse or progression may be driven by pre-existing subclones or by populations selected under pharmacologic pressure [14,15].

Cellular plasticity further compounds this genetic diversity. In STS, resistance does not depend exclusively on new mutations, but also on the ability of certain tumor cells to transition into persistent states that are less proliferative and more adaptable to therapeutic stress. This phenomenon overlaps with stemness-associated programs, in which populations with high ALDH activity, CD133 expression, and activation of transcriptional networks linked to SOX2, NANOG, and POU5F1 have been implicated. Although these markers are neither universal nor, by themselves, equivalent to a stable cancer stem cell population across all subtypes, the concept remains useful because it integrates several functional properties associated with resistance: relative quiescence, greater capacity for damage repair, metabolic adaptation, and the potential for tumor repopulation after drug exposure. In sarcoma, therefore, plasticity should be understood more as a dynamic functional state than as a fixed and homogeneous subpopulation [16].

This layered heterogeneity makes a linear model of STS resistance difficult to defend. Rather than a single universal mechanism, resistance in these tumors emerges as a systemic property of their underlying biological diversity. This principle is central to the rest of the review: refractoriness to chemotherapy, targeted therapies, or immunotherapy should not be interpreted as a uniform phenomenon but as the consequence of distinct molecular architectures that confer distinct vulnerabilities [9,10,11,14,15,16]. A histology-specific synthesis of molecular contexts, resistance programs, candidate biomarkers, and evidence levels is provided in Table 1.

## 3. Tumor Mechanisms of Resistance to Conventional Chemotherapy in STS: From Baseline Insensitivity to Adaptive Escape

### 3.1. Why It Is Useful to Distinguish Intrinsic Resistance from Acquired Resistance

In STS, resistance to conventional chemotherapy rarely depends on a single biological event. Rather, it reflects the convergence of multiple processes, including reduced intracellular drug accumulation, alterations in drug activation or detoxification, increased capacity to repair or tolerate DNA damage, and activation of survival programs that uncouple genomic injury from cell death [37,38]. This framework is particularly useful in STS because it allows the variable activity of anthracyclines, ifosfamide, and trabectedin to be interpreted within a common biological logic, while also explaining why responses differ across subtypes and among patients who appear to have received similar treatment strategies.

### 3.2. Intrinsic Resistance: Intracellular Drug Availability and Metabolic Processing

A first layer of resistance corresponds to intrinsic resistance, that is, pre-existing tumor characteristics that reduce sensitivity before therapeutic exposure. One of its classic mechanisms is a reduction in the effective intracellular drug concentration. In this context, ABC family transporters—particularly P-glycoprotein encoded by ABCB1 and MRP1 encoded by ABCC1—may promote efflux of anthracyclines and other cytotoxic agents, thereby reducing actual intratumoral exposure to treatment [37,38,39]. Functionally, this means that therapeutic failure may begin before cytotoxic damage reaches a biologically lethal threshold. In high-risk STS, the association between MRP1 overexpression and poorer clinical outcomes reinforces the relevance of this mechanism beyond the experimental setting [39].

Drug metabolism adds a further dimension, as it simultaneously modulates efficacy and toxicity. In the case of ifosfamide, this issue is particularly important because it is a prodrug whose bioactivation depends on cytochrome P450 enzymes, mainly CYP2B6 and CYP3A4, whereas competing detoxification pathways may reduce the generation of effective cytotoxic metabolites [38,40]. Accordingly, part of the clinical variability attributed to “resistance” may reflect differences in exposure to the active compound. Importantly, the prospective sarcoma pharmacokinetic study that used the erythromycin breath test as an in vivo CYP3A phenotyping assay found that this test did not correlate with ifosfamide pharmacokinetics or clinical toxicity, whereas 14-h concentrations of ifosfamide and its metabolites were associated with exposure and hematologic or renal toxicity [40]. Thus, host pharmacokinetic variation should be distinguished from tumor-cell-intrinsic drug resistance, and CYP3A activity measured by erythromycin breath testing should not be considered a validated biomarker of resistance.

### 3.3. Intrinsic Resistance: DNA Damage Response, Checkpoints, and the Apoptotic Threshold

A second layer of intrinsic resistance relates to the tumor’s capacity to detect, repair, and tolerate chemotherapy-induced damage. Both anthracyclines and ifosfamide generate complex DNA lesions, including replicative stress, strand breaks, crosslinks, and oxidative damage. Consequently, therapeutic sensitivity is modulated by the functional competence of pathways such as BER, NER, HR, and the Fanconi anemia/BRCA-like network [37,38]. Within this architecture, resistance does not depend solely on completely repairing the lesion, but also on maintaining cellular viability long enough to avoid replicative collapse and cell death.

Cell-cycle checkpoints and the apoptotic machinery are equally decisive. Even when genomic damage occurs, the tumor may survive if it can uncouple damage signaling from the execution of apoptosis. From this perspective, alterations in regulators of damage response and survival—including TP53, ATM, and signaling nodes associated with PI3K—may raise the apoptotic threshold and promote a chemoresistant phenotype [37,38]. Biologically, this means that intrinsic resistance in STS should not be understood only as “less drug entry,” but also as a greater capacity to absorb genotoxic stress without translating it into effective tumor cell death.

### 3.4. Acquired Resistance: Clonal Selection, Plasticity, and Therapeutic Persistence

Against this background of baseline insensitivity, acquired resistance may emerge during treatment exposure. In STS, this process is unlikely to be explained in every case by a single new alteration; rather, it probably reflects the progressive selection of cellular subpopulations better adapted to therapeutic stress [37,38]. Under pharmacologic pressure, clones with greater capacity for drug efflux, DNA repair, oxidative stress management, or post-damage survival may expand and become clinically dominant. Thus, relapse or progression after an initial partial response does not necessarily represent a different biology, but rather a selected and amplified version of the pre-existing heterogeneity.

Cellular plasticity also plays an important role in this transition. Some tumor cells may enter transient states of low proliferation or therapeutic persistence, surviving chemotherapy without actively proliferating and re-entering the cell cycle later [37,38]. This concept is particularly useful in STS because it connects cellular heterogeneity, damage adaptation, and tumor repopulation within a single dynamic model. In this sense, acquired resistance does not replace intrinsic resistance; it often expands it, reinforces it, and makes it clinically evident.

### 3.5. Alterations in Factors Promoting Invasion and Metastasis (EMT/MET Plasticity)

Unlike carcinoma, EMT/MET programs in sarcoma remain less clearly defined, because sarcomas already arise from mesenchymal lineages and do not follow the classical epithelial-to-mesenchymal paradigm. A more useful interpretation is that sarcoma cells may occupy metastable phenotypic states and, depending on lineage context and microenvironmental pressure, shift toward relatively epithelial-like or more mesenchymal programs [44,45]. This plasticity may contribute to invasion, metastatic competence, immune escape, stemness, and therapeutic tolerance.

In this setting, EMT/MET plasticity can be understood as a mechanism of phenotypic drug tolerance: cells adapt reversibly to therapeutic stress without requiring a fixed resistance mutation. Preclinical work has linked SNAIL-mediated programs to increased EZRIN and AKT signaling in rhabdomyosarcoma, while CCL21/CCR7–Slug signaling has been associated with EMT activation and invasive behavior in chondrosarcoma [46,47]. Although these data remain model-dependent, they reinforce the broader concept that lineage plasticity may shape pharmacologic resistance in selected sarcoma contexts.

### 3.6. Extracellular Vesicles

Extracellular vesicles, especially exosomes, have emerged as active mediators of tumor progression rather than passive biomarkers of disease biology. By transferring oncogenic proteins, microRNAs, metabolites, and other bioactive molecules, sarcoma-derived exosomes can activate survival programs, remodel the microenvironment, promote immune escape, and support stem-like or adaptive transcriptional states under therapeutic stress [48]. In liposarcoma, exosomal miR-25-3p and miR-92a-3p have been implicated in macrophage activation and tumor-promoting paracrine signaling, providing a mechanistic link between vesicle-mediated communication and progression [48].

Extracellular vesicles may also reinforce a pro-tumorigenic and immunosuppressive niche that limits the efficacy of chemotherapy, targeted therapy, and immunotherapy. Experimental delivery of TGF-β1 siRNA via microvesicles has shown tumor-suppressive effects in murine models, underscoring that vesicle biology is not only a mechanism of resistance but also a potential therapeutic platform [49]. These findings support the concept that exosomes are not merely biomarkers of aggressive disease but active drivers of phenotypic plasticity and multidrug resistance in sarcoma.

### 3.7. Mechanistic Expression in Anthracyclines, Ifosfamide, and Trabectedin

From an applied perspective, anthracyclines illustrate a multifactorial form of resistance in which drug efflux, variability in intracellular exposure, tolerance to topoisomerase II-induced damage, and the ability to buffer oxidative stress converge [37,38,39]. Their activity depends not only on the damage they produce, but also on the tumor’s inability to neutralize that damage. Ifosfamide, in turn, exemplifies how clinical efficacy may be shaped simultaneously by activation pharmacology, competing detoxification, and the tumor’s ability to tolerate crosslinks and replicative stress [38,40]. In both cases, resistance arises less from an isolated mechanism than from the interaction between pharmacology and the tumor’s molecular context.

Trabectedin represents a particularly valuable paradigm because it shows more clearly that sensitivity to a cytotoxic agent may depend on the status of the DNA repair machinery [50,51,52,53]. Its binding to the minor groove of DNA and its interaction with transcriptional and repair processes have made it clear that a functional NER system may contribute to its cytotoxic activity, whereas alterations in HR may modulate tumor vulnerability [50,51,52,53]. Trabectedin should therefore not be regarded simply as another active drug in STS, but rather as a proof of concept that response to conventional chemotherapy is conditioned by the tumor’s biological context. This observation is especially useful in a review focused on resistance, because it shows that the same repair circuits may confer resistance to some agents while creating vulnerability to others.

### 3.8. Biological and Clinical Implications of Chemoresistance in STS

Taken together, resistance to conventional chemotherapy in STS can be understood as arising from two complementary dimensions: baseline insensitivity, determined by intracellular drug availability, metabolism, damage-response capacity, and the apoptotic threshold; and evolutionary adaptation, driven by clonal selection and cellular persistence under therapeutic pressure [37,38]. This conceptual framework is more useful than a strictly “drug-by-drug” classification because it helps explain why different cytotoxic agents share common biological barriers, even if their clinical expression differs.

In addition, this framework creates a natural transition to the following sections. Once it is established that chemoresistance in STS depends on damage-repair networks, cellular plasticity, and biological context, it becomes easier to understand how these same principles later extend to resistance to targeted therapies, epigenetic modulators, immunotherapy, and dynamic biomarkers for disease monitoring [37,38,39,40,50,51,52,53].

## 4. Resistance to Targeted and Epigenetic Therapies: From Bypass Signaling to Lineage Adaptation

In STS, resistance to targeted therapies usually reflects a biology different from that observed in tumors governed by a single dominant oncogenic dependency. In many subtypes, tyrosine kinase inhibitors (TKIs) and antiangiogenic agents more often induce transient disease control than deep and sustained suppression of the tumor program. This pattern suggests that clinical progression depends less on an isolated secondary mutation and more on the tumor’s ability to activate compensatory programs, redistribute biological dependencies, and adapt to therapeutic stress [54,55].

### 4.1. Adaptive Escape from TKIs and Antiangiogenic Agents

In the setting of antiangiogenic agents and TKIs, resistance may emerge through adaptive escape pathways that restore proliferative, angiogenic, or survival signals without necessarily reactivating the original target itself. These mechanisms include activation of alternative proangiogenic pathways, recruitment of stromal support, increased pericyte coverage, adaptive hypoxia, and non-canonical forms of tumor vascularization [54,55]. Although many of these mechanisms have been described in the broader field of antiangiogenic oncology, their relevance to STS is particularly important, because agents such as pazopanib, regorafenib, and anlotinib likely exert their activity through simultaneous, but incomplete, blockade of several signaling networks.

This point matters because escape from antiangiogenic therapy in STS should not be understood simply as a “loss of sensitivity” to the drug, but rather as a redistribution of the tumor’s biological dependence toward alternative signals. Since these agents may simultaneously affect targets such as FGFR, PDGFR, MET, AXL, or intracellular nodes such as PI3K/AKT/mTOR, progression often reflects a systems-level readjustment rather than a single, clearly dominant resistance alteration [54,55].

### 4.2. Transcriptional Reprogramming and Lineage Plasticity

Bypass mechanisms are further compounded by transcriptional reprogramming, which likely represents one of the most relevant axes of adaptation under therapeutic pressure. In STS, an initial response to targeted therapy may be followed by a partial reduction in selective pressure that is nonetheless sufficient for the tumor to preserve viability through changes in gene expression programs, survival pathways, and phenotypic plasticity [56,57]. In this setting, progression does not necessarily imply reactivation of the same pathway that was initially inhibited, but rather the acquisition of a new cellular state capable of sustaining proliferation, survival, or invasion despite treatment.

This phenomenon is especially important in sarcomas defined by alterations in chromatin regulation or transcriptional control. In such contexts, therapeutic dependence resides not only in an active signaling pathway, but also in an epigenetic and lineage state that can be reconfigured. Resistance may therefore emerge not as a discrete genetic event, but as an adaptation of cellular state, with initial minimal residual persistence followed by later clinical progression even in the absence of a clearly identifiable secondary mutation [56,57].

### 4.3. Subtype-Specific Vulnerabilities and Heterogeneous Dependencies

A direct consequence of the above is that vulnerabilities in STS are profoundly subtype-specific. There is no single “targeted therapy for STS,” because this category encompasses biologically heterogeneous entities in which oncogenic dependencies are distributed unevenly across histologies. Some arise from specific fusions or alterations; others from lineage dependencies, particular microenvironmental configurations, or distinct epigenetic states [54,58]. This heterogeneity requires therapeutic results to be interpreted by subtype rather than through broad histologic aggregation, which tends to mix tumors with genuine vulnerabilities with others whose biology does not depend on the same therapeutic axis.

From this perspective, resistance reflects not only drug failure, but also a mismatch between the therapeutic mechanism and the tumor’s true biological dependency. In other words, part of the apparent resistance observed in STS trials may stem from the fact that the therapeutic target is relevant only to a limited subset of tumors within a broader histologic category [54,58].

### 4.4. SMARCB1-Deficient Epithelioid Sarcoma as a Useful, but Not Universal, Paradigm

A particularly illustrative example is SMARCB1-deficient epithelioid sarcoma. In this context, loss of INI1/SMARCB1 creates a relative epigenetic dependency on EZH2, which provided a convincing mechanistic rationale for the development of tazemetostat. The clinical activity observed with this inhibitor confirmed that this vulnerability is real and clinically actionable [21,22,23,56,57]. However, this case should be interpreted as a subtype-specific paradigm, not as a universal model that can be extrapolated to STS as a whole.

Indeed, even in this apparently well-defined setting, resistance may emerge through genetic and adaptive mechanisms that limit the depth or duration of response. This is relevant because it shows that, even when an epigenetic vulnerability is genuine, the biological dependency may still be incomplete, dynamic, or amenable to compensation. Accordingly, the conceptual value of the SMARCB1/EZH2 model does not lie in suggesting a general solution for STS, but in demonstrating that the efficacy of a targeted or epigenetic therapy depends on close alignment among subtype, chromatin state, and therapeutic mechanism [21,22,23,56,57].

### 4.5. Conceptual Implications for Resistance to Targeted Therapies in STS

Taken together, resistance to targeted and epigenetic therapies in STS can be organized into three complementary levels: adaptive pathway escape, transcriptional/epigenetic reprogramming, and subtype-specific dependencies that are only incompletely captured by treatment. This structure is more useful than an interpretation focused exclusively on secondary resistance mutations, because it better reflects the actual biology of most STS [54,55,56,57].

In addition, this framework has an important clinical implication: in STS, therapeutic failure is rarely explained by the mere loss of binding to a molecular target. More often, it reflects the tumor’s ability to reorganize its biology around alternative signals or to exit the cellular state that initially made the drug relevant. For this reason, the clinical activity of TKIs, antiangiogenic agents, and epigenetic modulators should always be interpreted through the lens of subtype, lineage context, and adaptive plasticity, rather than as though they belonged to a homogeneous therapeutic class [21,22,23,54,55,56,57,58].

### 4.6. Lessons from the KRAS Inhibitor-Resistance Landscape: A Conceptual Framework

Although derived from KRAS-driven carcinomas rather than STS, recent KRAS inhibitor studies provide a useful conceptual framework for adaptive resistance. Genome-wide CRISPR studies have identified multiple synthetic lethal dependencies, including the YAP/TAZ/TEAD axis, and have reinforced the rationale for mechanism-based combinations [59]. Complementary work in pancreatic cancer has shown that genetic and non-genetic resistance mechanisms can co-evolve through pathway reactivation, transcriptional plasticity, lineage-state transitions, and alternative survival programs [60]. In STS, these observations should be interpreted as a conceptual analogy—not as direct disease-specific evidence—but they support longitudinal monitoring, functional profiling, and combination strategies designed to anticipate parallel rather than single-mechanism escape.

## 5. The Tumor Microenvironment as a Mediator of Resistance

In STS, therapeutic resistance should not be understood solely as an intrinsic property of the tumor cell. A relevant component of the refractory phenotype emerges from the tumor microenvironment (TME), which shapes oxygen availability, drug access, metabolic plasticity, and the intensity of immune surveillance. Within this framework, resistance acquires an ecological dimension: the tumor survives not only because of its genomic alterations, but also because it resides in a niche that buffers the effects of treatment [61,62,63].

Hypoxia is one of the best-documented axes of this resistant niche in STS. Clinical studies have shown that hypoxic tumors are associated with poorer survival, and subsequent analyses confirmed the prognostic value of hypoxia-related markers, including HIF-1α, HIF-2α, GLUT-1, and CAIX [61,62]. From a biological standpoint, activation of HIF-1α and HIF-2α—encoded by HIF1A and EPAS1, respectively—promotes an adaptive program that includes increased glycolysis, extracellular acidification, angiogenic remodeling, and stress tolerance, all of which reduce the efficacy of chemotherapy, radiotherapy, and immunotherapy. In sarcomas, this metabolic reprogramming is not uniform across subtypes, but it reinforces a central principle: hypoxic pressure selects for cellular states that are more adaptive and less vulnerable to treatment [61,62,63].

This metabolic layer is further compounded by the physical barrier posed by the stroma. In STS, the extracellular matrix is not a passive scaffold but a dynamic component that influences tissue stiffness, vascular compression, drug diffusion, and survival signaling [64]. Increased tumor interstitial pressure may limit the penetration of systemic agents and contribute to regional exposure gradients, thereby facilitating the persistence of partially treated clones. Indeed, in refractory sarcomas, intratumoral interstitial pressure has been shown to correlate with tumor size, and vascular modulation can alter this parameter in at least a fraction of patients [65]. Thus, part of pharmacologic resistance in STS does not arise from absolute molecular insensitivity but from suboptimal intratumoral distribution of treatment [64,65].

The immunologic component of the TME also plays an active role in resistance. Across multiple sarcoma series, tumor-associated macrophages (TAMs) constitute a dominant population, often exceeding effective lymphocytic infiltration [66,67,68]. Moreover, their presence is commonly accompanied by expression of immunoregulatory checkpoints and by functional programs that promote immunosuppression, tissue remodeling, angiogenesis, and tumor repair [66]. In parallel, myeloid-derived suppressor cells (MDSCs), together with cytokine- and chemokine-mediated circuits, contribute to T-cell exclusion or dysfunction, reinforcing a “cold” immune state in many STS [69,70]. This landscape helps explain why a substantial proportion of sarcomas show only limited or inconsistent responses to immunotherapy given as monotherapy [66,67,68,69,70].

A particularly important point is that immunosuppression and angiogenesis do not operate as separate processes. Aberrant tumor vasculature, driven in part by signals such as VEGF-A—encoded by VEGFA—not only perpetuates hypoxia, but also hinders the trafficking and infiltration of effector immune cells [63,70]. In this way, pathologic angiogenesis promotes immune exclusion and consolidates a vicious cycle of resistance: hypoxia, metabolic reprogramming, vascular disorganization, and immunosuppression reinforce one another. Taken together, this framework supports a more contemporary view of resistance in STS: not all resistance is “tumor cell–intrinsic”; a substantial part is contextual and depends on the ecosystem in which that cell persists [63,64,65,66,67,68,69,70].

### Mesenchymal Stem Cell-like Features as the Putative Origin of Resistance in Sarcoma

Sarcoma biology is increasingly understood through the lens of mesenchymal progenitor states. Experimental and translational data support the concept that at least some sarcomas arise from primitive mesenchymal stromal/stem cells that accumulate successive oncogenic alterations [71]. These subpopulations can exhibit enhanced DNA damage repair, resistance to apoptosis, metabolic adaptability, and relative quiescence, enabling them to survive therapies that preferentially eliminate rapidly proliferating cells [72]. Stem-like sarcoma cells may also promote immune evasion and facilitate EMT/MET-associated phenotypic transitions, thereby sustaining intratumoral heterogeneity and adaptive resistance under therapeutic pressure. Experimental models further show that MDM2/CDK4 co-expression in transformed human mesenchymal stem cells can generate high-grade sarcoma phenotypes with dedifferentiated liposarcoma-like morphology, reinforcing the link between progenitor-state biology and therapy-resistant disease [18]. Collectively, these features support the hypothesis that cancer stem-like cells serve as a reservoir for disease persistence, relapse, and multidrug resistance in STS.

## 6. Immunotherapy in STS: Why Responses Are Often Limited

Unlike other solid tumors in which checkpoint blockade was incorporated relatively rapidly into systemic treatment, in soft tissue sarcomas (STS) the overall clinical activity of immunotherapy has been modest and inconsistent. This pattern should not be interpreted as an absolute absence of immunogenicity, but rather as the expression of a largely “cold” tumor group, with low baseline inflammation in many subtypes, limited sustained effector infiltration, and marked biological disparity across histology. In this context, studies of anti-PD-1 agents or combinations with anti-CTLA-4 have suggested that immunologic sensitivity does exist, but it is restricted to specific subgroups and cannot be extrapolated to STS as a whole [7,24,25,26,27].

One of the central challenges in the field has been the search for simple predictive biomarkers. Neither PD-L1 expression nor tumor mutational burden (TMB) has, on its own, shown a robust and reproducible ability to identify patients with STS who are likely to benefit. PD-L1 expression is heterogeneous across studies and subtypes, depends on different platforms and cutoffs, and often does not correlate linearly with clinical response. Similarly, TMB is generally low in most non-GIST STS and, although it may identify biological exceptions, it does not by itself capture the complexity of the tumor immune ecosystem. For this reason, the most recent reviews agree that isolated biomarkers are insufficient and that response prediction requires integrative models incorporating histology, immune architecture, and microenvironmental context [7,24,25].

Histology-specific heterogeneity of the immune infiltrate likely explains an important part of this clinical variability. In sarcomas, not only the quantity of infiltrating immune cells matters, but also their spatial organization and functional composition. Translational studies have shown that some tumors display relatively lymphocyte-poor microenvironments, whereas others exhibit more complex immune niches, including tertiary lymphoid structures (TLS), B-cell infiltration, and immune transcriptional signatures associated with better prognosis and a higher likelihood of benefit from immunotherapy. This finding has shifted the focus away from binary markers toward a more structural interpretation of the tumor immune phenotype [28,29,30].

Another relevant point is that immune resistance in STS does not appear to be confined to the PD-1/PD-L1 axis. Different studies have documented the expression of other checkpoints and inhibitory circuits—including VISTA, LAG-3, and TIGIT—whose contribution may vary across subtypes and cellular contexts. This reinforces the idea that failure of PD-1 blockade does not necessarily imply the absence of immune vulnerability, but may instead reflect redundancy of inhibitory pathways, lymphocyte exclusion, myeloid predominance, or a tumor architecture that is poorly permissive to a sustained effector response. Accordingly, STS represents a setting in which immunotherapy should be understood in terms of tumor ecology and immune adaptation, rather than as a single-target intervention [7,29,31].

Taken together, the available evidence suggests that the right question is not whether STS respond to immunotherapy, but which subtypes, which immune states, and which therapeutic combinations may convert exceptional sensitivity into a reproducible strategy. From this perspective, the main current limitation of the field is not only the modest magnitude of response, but also the absence of robust biomarkers that are validated across cohorts and useful in clinical practice. This framework justifies focusing the next discussion on strategies to overcome resistance, including rational combinations, biologically informed patient selection, and new approaches to dynamic monitoring [7,24,25,28,29,30].

## 7. Biomarkers for Sensitivity and Resistance Monitoring

Identifying useful biomarkers in STS faces a structural limitation: the marked biological heterogeneity of the group. Within the same diagnostic category, tumors with very different genomic architectures, transcriptional programs, immune interactions, and therapeutic dependencies coexist. Consequently, developing cross-cutting biomarkers has been more complex than in other solid tumors, and most available approaches remain more useful for biological stratification than for routine therapeutic selection applicable to all subtypes [37,73]. In this context, it is particularly important to distinguish between predictive baseline biomarkers—aimed at estimating sensitivity or resistance before treatment—and dynamic biomarkers, whose value lies in monitoring disease evolution under therapeutic pressure. The most relevant biomarkers for monitoring sensitivity and resistance in STS are summarized in Table 2.

### 7.1. Baseline Predictive Biomarkers

Pharmacogenomics was one of the earliest avenues explored to anticipate sensitivity or resistance. Among the most studied candidates are genes involved in drug transport and metabolism, as well as in DNA damage response and repair, including ABCB1, ABCC1, ERCC1, BRCA1, BRCA2, and SLFN11 [37]. However, in STS these biomarkers have not yet achieved sufficiently robust prospective validation to support routine incorporation into clinical practice. Their main contribution so far has been conceptual: they have shown that part of pharmacologic resistance may be conditioned by pre-existing biological states, rather than by a uniform phenomenon shared by all sarcomas [37]. This observation is relevant because it shifts the interpretation of resistance away from an exclusively acquired process toward a model in which baseline tumor biology already shapes, at least in part, therapeutic efficacy.

The evaluation of TOP2A and SIRT1 expression patterns further highlights both the clinical appeal and the limitations of biomarker-based stratification in high-risk STS. In patients treated with neoadjuvant anthracycline-based chemotherapy, increased TOP2A expression was associated with poorer overall survival, whereas higher SIRT1 expression correlated with more favorable outcomes. The combined profile of high SIRT1/low TOP2A showed additional prognostic value [75]. These data support further study of TOP2A/SIRT1 as candidate predictive or prognostic biomarkers, but they remain insufficient for routine clinical decision-making without prospective validation and assay standardization. Within this framework, the experience with trabectedin is especially illustrative. Unlike other systemic agents, its activity appears to be modulated by the DNA damage repair context and by certain chromatin configurations, which has encouraged the development of more mechanistically oriented biomarkers. In this regard, a gene signature associated with DNA repair has been linked to response to trabectedin in advanced STS, and more recent studies have suggested a possible role for HMGA1 as a modulator of sensitivity [52,74]. These findings are particularly interesting because they suggest a more specific interaction between the mechanism of action and tumor vulnerability, making them a useful example of biologically plausible biomarkers. However, they still do not justify broad clinical implementation, as they require external validation, analytical standardization, and prospective confirmation in independent cohorts [52,74].

Transcriptomics has substantially expanded the field by enabling the capture of complex biological states that are not apparent from a single molecular alteration. Signatures such as CINSARC have demonstrated mainly prognostic value, whereas other transcriptomic and immune-based approaches aim to identify tumors with a greater likelihood of therapeutic sensitivity [32,73]. At this point, it is important to underscore a key distinction: not every useful molecular biomarker is necessarily predictive. In STS, a substantial proportion of the available signatures provide primarily biological and prognostic information, but still do not reach a sufficient level of validation to guide individualized therapeutic decisions. Even so, these tools remain valuable because they refine the biological classification of the tumor and help identify subgroups with phenotypes that may be amenable to differentiated treatment strategies [32,73].

Among these approaches, the immunologic constant of rejection (ICR) and other signatures related to immune infiltration and adaptive immune activation have been particularly attractive, because they help distinguish between tumors with a microenvironment that is relatively more permissive to an effective immune response and those with exclusion or immunosuppressive phenotypes [32]. Their interest lies not only in describing the microenvironment, but also in approximating the tumor’s actual immune competence. Even so, their main current utility remains closer to biological enrichment and stratification than to fully validated individualized clinical prediction [32,73].

This point is especially relevant in immunotherapy. In STS, the limited overall activity of immune checkpoint inhibitors has driven the search for biomarkers more informative than PD-L1 expression or tumor mutational burden (TMB) assessed in isolation. In this context, the presence of tertiary lymphoid structures (TLS) represents one of the most consistent signals of biological enrichment for response, as it identifies tumors with a more organized and potentially more competent immune architecture [30,33]. Even in this setting, however, the message must remain cautious: TLS is a promising biomarker, but it is neither universal nor sufficient on its own to guide routine decisions across the full spectrum of STS [30,33]. Rather than serving as an isolated criterion for therapeutic selection, TLS seems better integrated within a broader interpretation of the tumor immune context.

### 7.2. Dynamic Biomarkers of Resistance

Unlike predictive baseline biomarkers, dynamic biomarkers seek to capture the evolution of the tumor throughout treatment. In this category, liquid biopsy—and in particular the analysis of circulating tumor DNA (ctDNA)—offers a particularly attractive opportunity for longitudinal monitoring of disease and resistance. Its appeal lies in the possibility of assessing early response, minimal residual disease, and relapse before overt radiologic progression becomes evident [19,20]. Conceptually, this represents an important shift: rather than merely estimating the initial risk of treatment failure, it would allow real-time observation of how the tumor responds, persists, or adapts under selective pressure.

The potential value of ctDNA in STS extends beyond simple quantification of tumor burden. In principle, it could also be used to detect molecular changes associated with emerging resistance and to follow clonal evolution during treatment. This is especially appealing in such a heterogeneous disease, where therapeutic pressure may favor the expansion of subclones with biological profiles different from those of the original tumor. In other words, liquid biopsy could provide not only a measure of disease burden, but also a dynamic window into the tumor’s changing biology. However, in sarcomas its performance is conditioned by several factors: tumor burden, the degree of tumor DNA shedding into plasma, the presence of trackable molecular alterations, and the sensitivity of the platform used [19,20]. For this reason, its current utility appears to be closer to selected settings—such as tumors with defined alterations or advanced high-grade disease—than to routine implementation across all contexts [19,20].

Mutational and transcriptomic signatures may also serve as dynamic biomarkers, capturing how the immune landscape of STS evolves under therapeutic pressure rather than providing only a static baseline snapshot [41]. The seven-gene SIGNIOS model integrates genes involved in immune activation, T-cell exhaustion, mesenchymal plasticity, and immunosuppressive regulation (CD86, CHI3L1, CXCL10, CXCL9, LAG3, NR4A1, and VCAM1). It may help identify patients more likely to benefit from PD-1 blockade and reflect adaptive resistance within the tumor microenvironment. Its validation across independent sarcoma cohorts supports its potential as both a prognostic and predictive tool for immunotherapy stratification in STS [42].

In addition, epigenetic strategies based on circulating methylated DNA are emerging, and these may be particularly useful in subtypes without a single, easily traceable genomic alteration. These approaches are conceptually attractive because they may overcome some of the limitations of conventional ctDNA in STS, especially in tumors with complex genomes or a low circulating tumor fraction. In this sense, circulating methylation could broaden the spectrum of tumors in which liquid biopsy is informative and, at the same time, provide a more stable signal in certain biological contexts [20,43]. Even so, these tools remain in a consolidation phase, with clear needs for multicenter validation, methodological harmonization, and the definition of clinically interpretable thresholds [20,43].

### 7.3. Current Limitations to Routine Implementation

The main barrier to translating these biomarkers into clinical practice is that the histologic and molecular heterogeneity of STS makes it difficult to extrapolate findings across subtypes [37,73]. A biomarker that appears promising in one specific subtype may lose value outside that context, which limits the possibility of constructing universal decision algorithms. This helps explain why many of the current advances are more solid as tools for biological enrichment than as broadly applicable clinical biomarkers.

#### Spatially Resolved Biomarkers and Resistance Niches

Spatially resolved profiling may help address a central limitation of current biomarker research in STS: distinguishing tumor-wide biological signals from resistance programs confined to discrete ecological niches. Bulk and single-cell analyses can identify immune, stromal, or transcriptional states, but they do not preserve the tissue architecture required to determine whether tumor cells, immune populations, cancer-associated fibroblasts, vascular structures, and hypoxic regions are spatially co-localized or segregated. This information is relevant because immune exclusion, stromal shielding, and localized clonal adaptation may be missed when biomarkers are interpreted without their spatial context [34,35,36].

An STS-specific illustration comes from a longitudinal synovial sarcoma case with acquired resistance to NY-ESO-1 TCR-T cell therapy. Spatial transcriptomic and proteomic profiling identified CTNNB1-high tumor regions that were negatively associated with T-cell surface proteins and antigen-presentation machinery, whereas bulk sequencing diluted these regionally restricted signals [17]. This was a cellular-therapy case, not anti-PD-1 resistance, but it demonstrates how spatially organized immune exclusion can generate actionable hypotheses for immunotherapy biomarker studies. In parallel, the TLS-selected PEMBROSARC cohort established that immune organization is clinically relevant: TLS-positive STS showed a 30% objective response rate with pembrolizumab plus low-dose cyclophosphamide, and plasma-cell abundance was associated with outcome [33].

Spatial transcriptomics, spatial proteomics, and high-plex molecular imaging can therefore complement liquid biopsy and conventional tissue biomarkers by identifying localized resistant clones, immune-excluded territories, myeloid-stromal barriers, and microenvironmental interactions that may be actionable but are obscured in bulk analyses. However, these technologies should currently be regarded as investigational. Their translation into treatment-selection tools will require prospective and longitudinal sampling, histology-specific cohorts, harmonized analytical workflows, reproducible scoring thresholds, and validation of their incremental clinical utility relative to standard molecular and pathological assessments [34,35,36].

This challenge is compounded by the limited standardization of many analytical platforms, particularly in transcriptomics, immunophenotyping, and liquid biopsy, as well as by variability in cutoffs and in the biological interpretation of results [19,20,32,43,73]. In practice, this lack of harmonization makes it difficult to compare studies, reproduce findings, and translate them into different clinical settings. Even when the biological signal is plausible, the absence of uniform analytical procedures may prevent a biomarker from achieving true clinical utility.

In the case of ctDNA and circulating methylated DNA, analytical sensitivity may be affected by the circulating tumor fraction, disease burden, and the existence of adequately traceable alterations [19,20,43]. This is particularly important in STS, where not all tumors release detectable amounts of tumor material into plasma and where the genomic architecture of the subtype directly influences the feasibility of molecular monitoring. Finally, cross-subtype validation in STS remains limited: much of the evidence derives from small cohorts, selected subtypes, or very specific biological settings, which restricts generalizability and complicates adoption as routine tools [19,20,37,43,73].

### 7.4. Translational Perspective

Taken together, biomarkers of sensitivity and monitoring in STS should currently be understood as tools for biological stratification and clinical hypothesis generation, rather than as mature instruments for universal routine use. Their greatest present strength does not lie in replacing clinical or histopathologic assessment, but in complementing it with a more refined reading of tumor vulnerability and biological evolution under treatment. In this sense, the field appears to be moving less toward a single biomarker and more toward integrated interpretive frameworks.

The most plausible future scenario does not appear to be an isolated biomarker, but combined models that integrate histology, molecular context, transcriptional state, immune architecture, and longitudinal monitoring through liquid biopsy [19,20,37,43,73,76]. This approach is particularly attractive in STS because it recognizes that therapeutic sensitivity does not depend on a single biological dimension, but on the interaction among multiple layers of information. Translated into the clinical setting, this would imply moving from therapeutic selection based almost exclusively on histology toward a more composite and dynamic biological stratification.

Even so, critical steps still remain before this vision can be widely adopted. These include the need for prospective validation, inter-platform standardization, demonstration of actionable clinical utility, and confirmation that these strategies truly improve therapeutic selection or monitoring compared with conventional approaches. For this reason, although the field is clearly promising, the current message must remain cautious: in STS, biomarkers of sensitivity and monitoring currently represent an expanding translational frontier, closer to precision medicine in development than to a fully established routine tool [19,20,37,43,73,76].

## 8. Emerging Strategies to Reverse or Prevent Resistance

**Figure 1:**
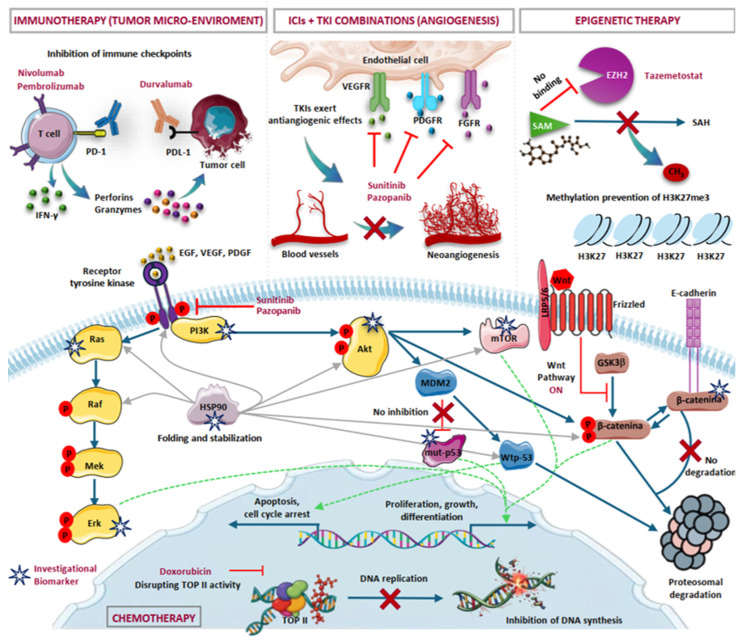
Evidence-Based Development of Targeted Therapies: Translational Advances in Soft-Tissue Sarcomas. Emerging therapeutic strategies derived from translational advances for the treatment of therapy-resistant soft-tissue sarcomas. Doxorubicin induces DNA damage and inhibits DNA replication, thereby interfering with cell division. Immune checkpoint inhibitors targeting the PD-1/PD-L1 axis promote T-cell activation and enhance antitumor immunity. Tyrosine kinase inhibitors (TKIs) act as antiangiogenic agents, promote vascular normalization, and inhibit VEGFR-, FGFR-, and PDGFR-mediated signaling pathways implicated in therapeutic resistance. It was missing: The client proteins of the Hsp90 chaperone are also represented by gray arrows. In addition, combination strategies incorporating epigenetic modulators are depicted as approaches to restore the expression of tumor suppressor genes.

In advanced STS, reversal of resistance is unlikely to be achieved through a single pharmacologic intervention. Rather, the current direction of the field favors rational combinations that target complementary vulnerabilities: the tumor cell, its adaptive circuits, and the microenvironment that buffers therapeutic effects. From this perspective, the selection of combinations should not rely solely on aggregate clinical activity, but on an explicit biological hypothesis, ideally anchored in histology, molecular context, and the tumor immune state [77]. The translational advances described throughout this review have fostered the development of novel therapeutic strategies for therapy-resistant soft tissue sarcomas, which are summarized in Figure 1. The main strategies currently being explored to reverse or prevent resistance in STS are summarized in Table 3.

### 8.1. Rational Mechanism-Based Combinations

A first avenue is to develop combinations grounded in complementary biological mechanisms rather than in the simple overlap of active agents. Within this framework, combining chemotherapy with immunotherapy is one of the most extensively studied paradigms. The rationale is well known: some cytotoxic agents may increase antigen release, tumor immunogenicity, and microenvironmental remodeling, thereby creating a window of increased susceptibility to immune checkpoint inhibitors (ICIs). However, in STS, this approach should not be treated as a universal principle. The study of doxorubicin plus pembrolizumab in advanced sarcoma suggested clinical activity and reinforced the hypothesis of synergy, but the magnitude of benefit remains heterogeneous across histologies and does not yet justify broad extrapolation to all STS [78]. In other words, the biological plausibility of the combination is real, but its efficacy appears to depend on the tumor context in which it is applied.

A similar logic underlies the combination of ICIs with antiangiogenic agents or tyrosine kinase inhibitors (TKIs). In this setting, the hypothesis is not simply to add two active classes, but to modulate the microenvironment so that tumor vasculature becomes less dysfunctional, myeloid immunosuppression decreases, and immune-cell trafficking into the tumor improves. Both nivolumab plus sunitinib and durvalumab plus pazopanib have shown that this vascular and immunologic modulation can translate into responses in specific subgroups, but they have also made clear that sensitivity depends on histologic and biological context, not only on drug mechanism [79,80]. For this reason, these combinations appear more plausible in subtypes with angiogenic dependence or in tumors whose microenvironment is susceptible to vascular reprogramming, rather than as cross-cutting regimens applicable across the full STS spectrum.

A third group of rational combinations includes targeted therapies together with epigenetic or transcriptional modulators. Here, the aim is not only to inhibit a dominant oncogenic pathway, but also to prevent the adaptive reprogramming that enables therapeutic escape. This approach is particularly attractive in subtypes in which lineage identity and dependence on epigenetic programs are part of the resistant phenotype. Conceptually, these strategies respond to a central idea: resistance does not always emerge through classic secondary mutations, but also through changes in cellular state and transcriptional plasticity. Even so, outside subtype-specific contexts, the clinical evidence remains stronger as proof of principle than as a therapeutic standard [77].

### 8.2. Antibody–Drug Conjugates

Antibody–drug conjugates (ADCs) represent a major recent therapeutic advance in oncology. They couple a monoclonal antibody directed against a tumor-associated antigen to a cytotoxic payload through a chemical linker, making therapeutic activity dependent, in large part, on target expression and internalization. In STS, systematic profiling has identified multiple ADC targets with subtype-specific overexpression, including 41 targets overexpressed in at least 25% of samples in at least one histologic subtype [82]. Among these, B7-H3 is particularly relevant because it is broadly expressed across STS and may provide a targetable surface antigen for biomarker-driven therapy [83].

HS-20093 targets B7-H3 (CD276), a surface protein frequently overexpressed in sarcomas and associated with immune evasion, tumor progression, and adverse prognosis. By selectively delivering a topoisomerase I inhibitor payload to B7-H3-expressing tumor cells, this ADC strategy aims to increase intracellular drug exposure while limiting systemic toxicity. From a resistance perspective, ADCs may partially bypass classical mechanisms of chemoresistance, including poor tumor penetration, intratumoral heterogeneity, and drug-efflux pathways, supporting their emerging role as biomarker-driven therapy in refractory STS [91].

### 8.3. Biomarker-Guided Strategies

If resistance in STS is not uniform, its reversal should not be approached in an undifferentiated way. A second key strategy, therefore, is to select patients according to identifiable biological vulnerabilities. In this context, biomarker-guided approaches add a layer of precision, enabling a shift from empirical combinations to biologically enriched ones. Rather than asking which regimen shows some activity in sarcoma, the relevant question is in which subtype, with which molecular program, and under which biological condition that combination has the greatest plausibility of benefit.

Molecular profiling is beginning to identify therapeutic signals, although most remain better suited for enrichment and hypothesis generation than for routine treatment selection. Tumor profiling studies have suggested genomic and transcriptomic correlates of response to pazopanib, while prospective platforms such as MULTISARC illustrate attempts to integrate molecular profiling into therapeutic design in advanced disease [81,84,86]. Although these findings are not yet universally reproducible, their importance is both conceptual and methodological: they show that biological enrichment may be more informative than simple histologic aggregation.

In addition, a biomarker-guided strategy should not be limited to baseline patient selection. Ideally, it should also incorporate response-adapted adjustment, that is, the possibility of modifying therapeutic direction as signals of sensitivity or escape emerge. This connects directly with the need to combine static biomarkers—genomic, transcriptomic, or immune—with dynamic markers that allow longitudinal tracking under treatment. In STS, that model is still under construction, but it likely represents one of the most reasonable paths to avoid both therapeutic overexposure and persistence with biologically ineffective combinations [77,81,84,86].

### 8.4. Emerging Vulnerabilities

A third axis consists of exploiting emerging vulnerabilities that may transform resistance into therapeutic fragility. Within this group, DNA damage response (DDR) targeting and synthetic lethality occupy a central place. In non-GIST STS, this field remains promising but immature. The underlying idea is that certain tumors may depend on compensatory DNA repair networks, such that blocking nodes such as ATR or PARP—especially in combination with chemotherapy or radiotherapy—may increase tumor vulnerability [85,87]. The appeal of this strategy lies in the fact that it does not simply seek to intensify treatment, but rather to exploit a specific dependency of the resistant tumor.

Preclinical data show that ATR inhibition can sensitize STS cells to chemotherapy even beyond the context of ALT, while recent molecular analyses suggest that DDR alterations are not uniform across histology and may also interact with immune biomarkers [85,87]. Even here, however, conceptual overextension should be avoided. For now, the value of synthetic lethality in STS appears more convincing as a paradigm for stratification than as an approach ready for broad adoption. Put differently, it is a strategy with strong biological plausibility, but its clinical translation still requires more robust biomarkers and more reproducible validation [85,87].

Patient-derived organoids and other functional precision platforms should also be considered among these emerging vulnerabilities. Their main appeal lies in allowing drug sensitivity and combination testing in systems that are closer to the individual tumor than conventional cell lines. In theory, this could be particularly useful for prioritizing salvage treatments or exploring resistance mechanisms that are not evident from isolated molecular characterization. However, in sarcoma important limitations remain: variable establishment rates, incomplete representation of the microenvironment, insufficient standardization, and difficulty scaling their use into routine clinical practice [89,90]. For this reason, their current value is primarily translational and hypothesis-generating, although they could become more relevant if successfully integrated with molecular biomarkers and adaptive trial designs.

### 8.5. Methodologic Redesign of Clinical Development

All of the above has an unavoidable methodological consequence: if resistance in STS is histology-dependent, biologically contextual, and dynamically modulated, then clinical trials must be as well. Broad designs that group together biologically disparate entities tend to dilute signal and make it more difficult to identify truly sensitive subgroups. In this context, adaptive designs, biomarker-enriched cohorts, and histology-specific studies likely represent a more informative path than traditional trial schemas [86,88].

This need is not merely technical, but conceptual. The question should no longer be only whether a combination “works in STS,” but in which subtype, under which escape hypothesis, with which selection biomarker, and with which monitoring strategy. For this reason, the methodological redesign of clinical development should not be viewed as an accessory issue, but as a prerequisite for translating the biology of resistance into interpretable therapeutic signal. Increasingly, this will require integrating dynamic biomarkers into the trial design itself, so that response, persistence, and escape can be documented longitudinally rather than only at the end of a therapeutic sequence [86,88,89].

In rare and biologically divergent STS, this approach also allows a more efficient allocation of clinical and biological resources. Although it entails greater logistical complexity, sample-size challenges, and dependence on expert centers, it probably offers a far more favorable balance between biological plausibility and clinical learning than undifferentiated designs [86,88]. In other words, if the goal is to reverse or prevent resistance, it is not enough to innovate in drugs; we must also innovate in how they are tested.

Taken together, the reversal or prevention of resistance in STS does not appear to be moving toward a universal monotherapy, but rather toward a strategy based on combination, context, and selection. Rational mechanism-based combinations, biomarker-guided approaches, exploitation of emerging vulnerabilities, and methodological redesign of clinical development should be understood as complementary components of the same therapeutic logic, not as separate and independent lines of work [77,78,79,80,81,84,85,86,87,88,89,90].

The practical implication of this view is clear: the problem of resistance in STS will not be solved by identifying a single “more active” agent, but by constructing therapeutic frameworks that align tumor biology, mechanistic hypothesis, patient selection, and longitudinal monitoring. From this perspective, the central question is no longer what is being attempted, but how reversal of resistance should be conceptualized in a family of tumors in which heterogeneity is not the exception, but the defining feature.

## 9. Conclusions

**Figure 2:**
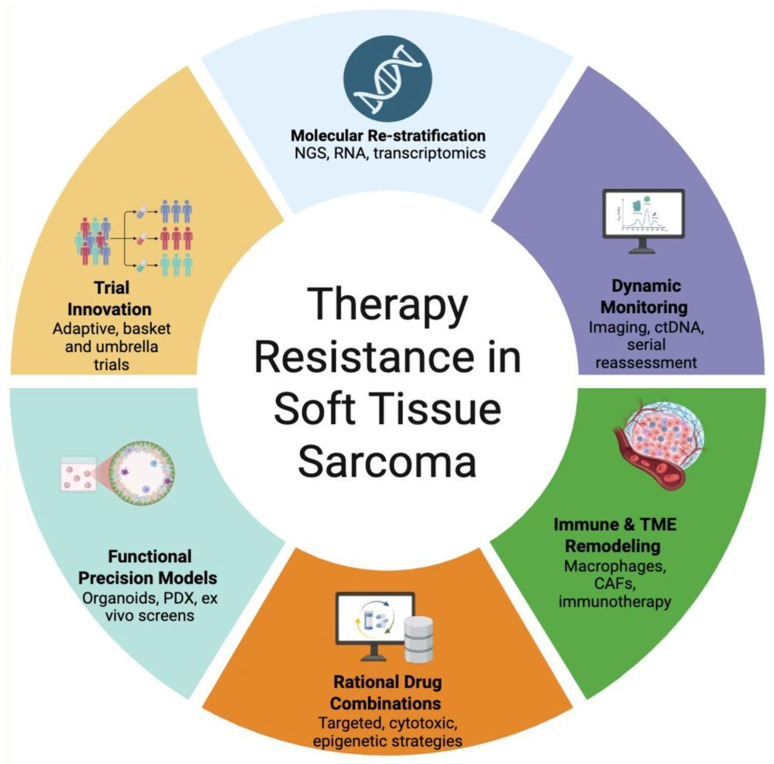
Translational framework for therapy-resistant soft tissue sarcoma. The figure summarizes key strategies to address therapeutic resistance in STS, including molecular re-stratification, serial monitoring, immune–microenvironment remodeling, rational combinations, functional precision models, and adaptive trial designs.

Therapeutic resistance in adult non-GIST soft tissue sarcomas (STS) should be understood as a multilevel phenomenon. It does not arise solely from genomic alterations acquired during treatment exposure, but also from epigenetic programs, persistent cellular states, phenotypic plasticity, metabolic adaptation, interaction with the tumor microenvironment, and circuits of immune exclusion or suppression [33,37,77]. This framework is important because it avoids overly reductionist interpretations: in STS, progression under treatment rarely reflects a single dominant mechanism and more often represents the convergence of several layers of biological resistance. In this sense, Figure 2 summarizes key strategies to address therapeutic resistance in STS.

Fusion-defined lineage programs and spatially organized immune-excluded niches are particularly relevant examples of mechanisms that cannot be fully captured by histology or bulk molecular testing alone [12,13,17,34,35,36].

Accordingly, therapeutic responses should always be interpreted by subtype, rather than as if STS constituted a biologically uniform entity. The same intervention may have different implications depending on histology, molecular architecture, immune context, and the tumor’s functional dependency. This principle applies to conventional chemotherapy, targeted therapies, immunotherapy, and emerging combinations alike, and it helps explain why extrapolation across subtypes is a recurrent source of inconsistent results and overstated therapeutic expectations [77,81,85,87].

Consequently, the future of the field will likely not lie in identifying a purported universal “master mechanism” of resistance, but in integrating histology, tumor biology, spatially resolved interpretation of the immune microenvironment, and dynamic longitudinal monitoring. This approach will require coordinated use of expert histologic classification, molecular and transcriptomic characterization, liquid biopsy where feasible, functional studies, and prospective histology-specific clinical trials designed to test biologically grounded combinations rather than purely empirical therapeutic sequences [17,19,20,34,35,36,85,88,89,90].

## Figures and Tables

**Table 1:** Histology-specific resistance programs, candidate biomarkers, and translational implications in adult soft tissue sarcoma.

Histotype/Molecular Context	Systemic Therapeutic Context	Dominant Reported Resistance Programs	Candidate Biomarkers/Readouts	Evidence Level and Translational Implication
Synovial sarcoma/SS18::SSX	Cytotoxic therapy; TCR-T and immune checkpoint strategies under investigation.	Fusion-directed chromatin and lineage programs; CTNNB1-associated regional immune exclusion in a longitudinal TCR-T resistance case.	SS18::SSX; CTNNB1 activation; spatial T-cell and antigen-presentation distribution.	Clinical–translational, hypothesis-generating. Not a validated predictor of anti-PD-1 resistance [12,17].
Myxoid liposarcoma/FUS::DDIT3 or EWSR1::DDIT3	Cytotoxic therapy including trabectedin; immunotherapy investigational.	Fusion-associated regulation of chromatin, extracellular matrix, immune-response-associated pathways, and metabolism in preclinical models.	Fusion transcript; extracellular-matrix, HLA, and spatial immune programs.	Preclinical/early translational. No validated evidence that fusion quantity changes drive therapeutic resistance [13].
Dedifferentiated liposarcoma/MDM2-CDK4 amplification	Anthracycline-based therapy; MDM2/CDK4- and DNA-repair-directed strategies under evaluation.	Genomic complexity, biological redundancy, subclonal diversification, and DNA-damage-response adaptation.	MDM2/CDK4 status; DNA-repair signatures; trackable ctDNA when feasible.	Translational. Biomarker-guided selection remains investigational [9,10,11,18,19,20].
Epithelioid sarcoma/SMARCB1 loss	EZH2 inhibition with tazemetostat; chemotherapy in advanced disease.	Incomplete or compensated epigenetic dependency despite a biologically actionable chromatin vulnerability.	INI1/SMARCB1 loss; EZH2-related context.	Clinical subtype-specific paradigm; resistance mechanisms require prospective characterization [21,22,23].
Undifferentiated pleomorphic sarcoma/complex genome	Chemotherapy, immune checkpoint inhibition, and antiangiogenic approaches.	Myeloid immunosuppression, immune exclusion, heterogeneous immune activation, and adaptive microenvironmental escape.	TLS, B-cell/plasma-cell infiltration, ICR, and spatial immune organization.	Clinical–translational; immune biomarkers enrich but are not universal treatment selectors [17,24,25,26,27,28,29,30,31,32,33,34,35,36].
Leiomyosarcoma/complex genome	Chemotherapy, antiangiogenic therapy, and selected investigational combinations.	Complex genomic background, DNA-repair and apoptotic-threshold variation, and adaptive pathway bypass.	DNA-repair signatures; dynamic ctDNA or methylated-DNA approaches where informative.	Exploratory/translational; requires histology-specific validation [19,20,37,38,39,40,41,42,43].

**Table 2:** Relevant biomarkers of sensitivity and resistance monitoring in STS.

Biomarker/Strategy	Sample Type/Platform	Potential Clinical Context	Main Finding	Current Implementation	Main Limitations
ABCB1/P-glycoprotein, ABCC1/MRP1, and other drug transport/metabolism genes	Tumor tissue; IHC, RT-qPCR, transcriptomics	Prediction of response or resistance to anthracyclines and ifosfamide	Biologically plausible association with drug efflux, altered intracellular exposure, and reduced cytotoxic activity [37]	Not routine	High heterogeneity by subtype, assay, treatment context, and cut-off definition [37]
DDR-related genes and signatures, including ERCC1, BRCA1, BRCA2, SLFN11, and related repair pathways	Tumor tissue; NGS, gene expression	Enrichment for sensitivity or resistance to DNA-damaging agents, particularly trabectedin	Consistent biological rationale linking DNA repair capacity with treatment response, but without universal clinical standardization [37,52]	Exploratory/translational	Lack of prospective validation; strong dependence on histologic subtype, drug mechanism, and assay [37,52]
DNA repair-associated gene-expression signature for trabectedin	Tumor tissue; gene-expression profiling	Selection of patients with a higher likelihood of benefiting from trabectedin	Associated with response and clinical outcomes in advanced STS [52]	Not routine	Requires independent external validation, assay harmonization, and prospective clinical confirmation before implementation [52]
HMGA1	Tumor tissue; gene/protein expression	Candidate biomarker of trabectedin sensitivity	Suggests a relationship between chromatin organization, HMGA1 biology, and trabectedin response [74]	Exploratory	Early evidence; not validated as a standalone biomarker for treatment selection [74]
TOP2A and SIRT1 expression profile	Tumor tissue; IHC/expression-based assessment	Candidate predictive or prognostic biomarker in anthracycline-treated high-risk STS	Expression patterns may correlate with outcomes after neoadjuvant anthracycline-based chemotherapy [75]	Exploratory	Requires prospective validation, standardized scoring, and clarification of whether the signal is predictive, prognostic, or both [75]
CINSARC	Tumor tissue; transcriptomic signature	Biological risk stratification	Stronger as a prognostic classifier than as a treatment-predictive biomarker [32,73]	Investigational/selective use	Does not define sensitivity to a specific systemic therapy by itself [32,73]
ICR and immune transcriptomic signatures	Tumor tissue; transcriptomics	Identification of tumors with a more active immune microenvironment	Refines prognostic assessment and may enrich for immune-responsive phenotypes [32,73]	Investigational	Reproducibility across cohorts, platforms, and histologic subtypes remains incomplete [32,73]
Intratumoral tertiary lymphoid structures (TLS)	Tumor tissue; histology, IHC, immune profiling	Selection or enrichment for immunotherapy benefit	In the TLS-positive PEMBROSARC cohort, pembrolizumab plus cyclophosphamide achieved an objective response rate of 30% [33]	Promising tissue biomarker; not universal	Detection methods vary across cohorts; predictive value depends on histologic and immune context [30,33]
Blood-based immunotherapy biomarkers, including cytokines, peripheral immune-cell profiles, and cfDNA	Peripheral blood; soluble markers, immune profiling, cfDNA assays	Monitoring and enrichment for response to immune checkpoint inhibitors	Emerging field, but no robust, reproducible blood-based biomarker has been established in STS [76]	Not routine	Limited standardization, small cohorts, scarce prospective validation, and uncertain clinical actionability [76]
Personalized ctDNA or ctDNA based on trackable genomic alterations	Plasma; NGS, ddPCR	Minimal residual disease, response assessment, early relapse detection, and resistance monitoring	Allows potential longitudinal monitoring of tumor burden and clonal evolution in selected STS contexts [19,20]	Investigational	Low tumor-DNA shedding in some subtypes; requires trackable alterations, sensitive platforms, and standardized thresholds [19,20]
Circulating methylated DNA	Plasma; ddPCR/epigenetic assays	Disease monitoring and tumor-burden assessment, particularly in high-grade or advanced sarcomas	Promising strategy that may be less dependent on a single genomic alteration than conventional ctDNA [43]	Advanced proof of concept	Requires multicenter prospective validation, pre-analytical standardization, and clinically interpretable cut-offs [43]

**Table 3:** Emerging strategies to reverse or prevent therapeutic resistance in STS.

Strategy	Main Biological Rationale	Representative Evidence	Setting with Greatest Plausibility	Current Limitations
Chemotherapy plus immune checkpoint inhibition	Cytotoxic therapy may increase antigen release, tumor immunogenicity, and microenvironmental remodeling.	Doxorubicin plus pembrolizumab in advanced sarcoma [78]	Selected advanced STS with an inflamed baseline state or histologies with immune sensitivity.	Heterogeneous activity across histologies; no universal standard-of-care role.
Immune checkpoint inhibition plus antiangiogenic agents/TKIs	Vascular normalization may improve immune-cell trafficking and attenuate myeloid immunosuppression.	Nivolumab plus sunitinib [79]; durvalumab plus pazopanib [80]	Tumors with angiogenic dependence and a microenvironment potentially amenable to vascular-immune modulation.	Response appears context-dependent; toxicity and optimal sequencing remain uncertain.
Targeted therapy plus epigenetic or transcriptional modulation	Co-targeting adaptive circuitry and lineage or chromatin-state programs may limit reprogramming escape.	Subtype-specific epigenetic paradigms and translational rationale [77,81]	Fusion-driven or chromatin-altered histologies with a defined biological dependency.	Clinical evidence remains fragmented and often preclinical.
Antibody-drug conjugates	Selective delivery of cytotoxic payloads may circumvent some limitations of conventional systemic exposure.	Target-expression profiling and B7-H3-directed approaches [82,83]	Tumors with sufficiently homogeneous, safely targetable antigen expression.	Antigen heterogeneity, on-target off-tumor toxicity, and limited sarcoma-specific efficacy data.
DNA damage response and synthetic lethality	Exploits tumor dependence on DNA repair, replication stress, or compensatory survival pathways.	Trabectedin–olaparib combinations and related DDR strategies [84]	Molecularly selected tumors with plausible repair vulnerabilities.	Predictive biomarkers and clinical selection algorithms remain immature.
Biomarker-guided treatment selection	Integrates genomic, transcriptomic, immune, and functional evidence to enrich for biologically matched therapy.	Molecular screening platforms such as MULTISARC [85]	Advanced disease managed in specialist centers with molecular tumor-board interpretation.	Limited prospective proof that matching improves outcome across unselected STS.
Adaptive and histology-specific trial designs	Aligns trial architecture with rare, biologically divergent histologies and evolving resistance states.	Histology-specific phase II strategies and benchmark analyses [86,87,88]	Rare subtypes and settings in which broad STS aggregation obscures signal.	Recruitment, sample size, and regulatory/logistical complexity.
Organoids and functional precision platforms	Uses ex vivo drug testing to complement genomic inference and identify patient-specific vulnerabilities.	Sarcoma organoid and functional precision studies [89,90]	Selected patients when sufficient viable tumor tissue and rapid turnaround are feasible.	Standardization, scalability, and prospective clinical utility remain unresolved.

## Data Availability

No new data were created or analyzed in this study. Data sharing is not applicable to this article.
